# Cilengitide Inhibits Attachment and Invasion of Malignant Pleural Mesothelioma Cells through Antagonism of Integrins αvβ3 and αvβ5

**DOI:** 10.1371/journal.pone.0090374

**Published:** 2014-03-03

**Authors:** Ngan Ching Cheng, Nico van Zandwijk, Glen Reid

**Affiliations:** Asbestos Diseases Research Institute, University of Sydney, Concord, New South Wales, Australia; Thomas Jefferson University, United States of America

## Abstract

Malignant pleural mesothelioma (MPM) is an almost invariably fatal, asbestos-related malignancy arising from the mesothelial membrane lining the thoracic cavities. Despite some improvements in treatment, therapy is not considered curative and median survival following diagnosis is less than 1 year. Although still classed as a rare cancer, the incidence of MPM is increasing, and the limited progress in treating the disease makes the identification of new therapies a priority. As there is evidence for expression of the integrins αvβ3 and αvβ5 in MPM, there is a rationale for investigating the effects on MPM of cilengitide, a synthetic peptide inhibitor of integrin αv heterodimer with high specificity for αvβ3 and αvβ5. In mesothelial cells (MC) and 7 MPM cell lines, growth inhibition by cilengitide was associated with the expression level of its target integrins. Furthermore, cilengitide caused cell detachment and subsequent death of anoikis-sensitive cells. It also suppressed invasion of MPM cells in monolayer and three-dimensional cultures. Gene knockdown experiments indicated that these effects of cilengitide were, at least partly, due to antagonism of αvβ3 and αvβ5.

## Introduction

Malignant pleural mesothelioma (MPM), originating in the mesothelial lining of the thoracic cavities, is strongly associated with exposure to asbestos [1]–[3]. The mesothelium is particularly susceptible to asbestos [4]. MPM is a highly invasive tumour with poor prognosis and resistance to therapy. Hence, the search for more effective treatment is a priority.

Integrins are a class of cell adhesion molecules mediating cell-cell and cell-matrix interactions. They are heterodimeric receptors for extracellular matrix (ECM). Combinations of 18 α and 8 β subunits form the 24 members of the integrin family. They bind to extracellular ligands including collagens, laminins, fibronectins, fibrinogen and vitronectin, linking the ECM to the cytoskeleton and thus creating a scaffold for tissue architecture. In addition to this function, integrins act as cell sensors that signal, for example, through activation of focal adhesion kinase (FAK) to regulate cell shape, attachment, proliferation, survival, motility, apoptosis and differentiation [5].

Integrin αvβ3 is the most versatile member of this family, having broad substrate specificity allowing the cell to react with many matrix proteins in its environment, eliciting a wide range of intracellular signals [6]. Angiogenesis is required to sustain tumour growth from hyperplasia to neoplasia [7], and *in vivo*, αvβ3 is expressed on angiogenic endothelial cells [8]. In a variety of tumour models, antagonists of αvβ3 reduced the number of tumour blood vessels, leading to tumour regression and suppressed invasion [9], [10]. Thus, integrin αvβ3 is an attractive target for cancer therapy.

Eight integrins recognise the arginine-glycine-aspartic acid (RGD) tripeptide motif in their ligands. Cilengitide is a cyclic RGD peptide that selectively inhibits the integrin αv subunit, which can form heterodimers with subunits β1, β3, β5, β6, or β8. Cilengitide has high specificity for αvβ3 and αvβ5 integrins and showed anti-angiogenic effect in corneal or chorioallantoic membrane models [11]–[13]. In addition, it inhibits growth and promotes apoptosis of tumour cells, such as glioblastoma, that express these integrins [14], [15]. Cilengitide showed efficacy in preclinical cancer models and phase I clinical trials [16], [17] and has gone on to phase II trials for cancers including glioblastoma, melanoma, prostate, breast, lung and head and neck cancers [18].

Aberrant integrin expression, notably of αvβ3, has been associated with tumour invasion and metastasis [19]. This is relevant to MPM as local invasion is the main cause of death in patients [20]. Analysis of integrin subunits has shown high expression of β1, α6 and αv in MPM specimens and cell lines [21]–[23]. Furthermore, two integrin αv ligands are thought to play a role in MPM: osteopontin, as a biomarker [24], [25] and vitronectin, reported to enhance the internalisation of asbestos by mesothelial cells [26]. Aberrant integrin αv expression thus appears significant for MPM and cilengitide may have clinical potential for its treatment.

In this study, we have analysed the expression of αv integrin subunits and receptors and we compared the effects of gene knockdown with integrin inhibition by cilengitide in MC and MPM cell lines. Cilengitide caused detachment of some MPM cells and inhibited proliferation of those that were susceptible to anoikis. Moreover, it suppressed invasiveness of monolayer and three-dimensional MPM spheroid cultures. These effects were partially reproduced by down-regulation of β3 and β5 integrins by gene knockdown, consistent with the actions of cilengitide.

## Results

### Expression of cilengitide target integrin subunits and receptors in mesothelial cells and MPM cell lines

**Figure 1:**
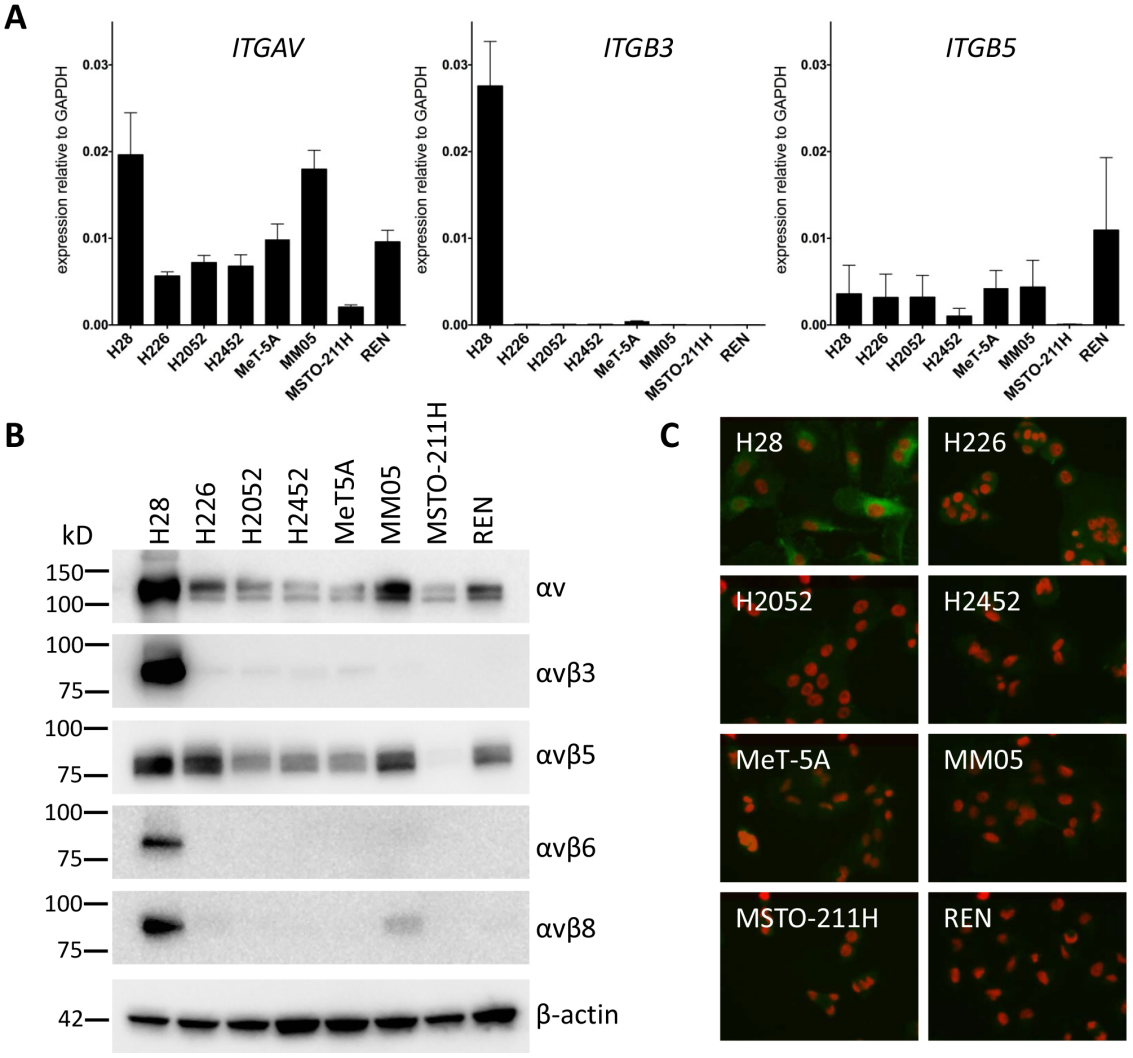
Expression of the integrin subunits and heterodimers that are targeted by cilengitide. (A) Relative mRNA levels of cilengitide target integrin subunits were measured by real-time qPCR and normalised to the Glyceraldehyde 3-phosphate dehydrogenase (*GAPDH*) control gene expression. Each cell line was analysed at least twice with two independent RNA samples. (B) Western blot analysis of αv integrins under non-reducing condition. The β-actin protein serves as loading control. Molecular sizes are indicated on the left of the blots. (C) Immunofluorescence of αvβ3 in MPM cells. The cells were incubated with a rabbit monoclonal antibody against αvβ3 and detected with an Alexa Fluor 488-conjugated secondary antibody.

The cilengitide target αv is encoded by the *ITGAV* gene. Its expression was determined by qPCR in non-malignant mesothelial cells MeT-5A and 7 MPM cell lines and found to be at moderate levels in most of them (Figure 1A). Of the genes encoding its major beta integrin partners, *ITGB5* was expressed moderately in most cells and *ITGB3* at low levels except in H28 cells, where it was high. Of the other beta partners forming integrins recognized by cilengitide with lower affinity, *ITGB1* was expressed abundantly, while *ITGB6* and *ITGB8* were expressed at low to undetectable levels (not shown). The MSTO-211H cell line had generally low expression of all cilengitide target genes.

Expression of the corresponding heterodimeric integrin proteins was evaluated using western analysis with antibodies specific for αv, αvβ3, αvβ5, αvβ6 and αvβ8 [27]. Results were consistent with the qPCR analysis, with all cells showing substantial αv expression, the most in H28 cells, followed by MM05 (Figure 1B and Table 1). Strong αvβ3 expression was seen in the H28 cells but it was barely detected in other cells. All cell lines, except MSTO-211H, expressed moderate levels of αvβ5. Expression of αvβ6 and αvβ8 was weak, except in H28 cells. Analysis of αvβ1 expression is challenging as the β1 subunit can form complexes with 12 different αsubunits, and specific antibodies for αvβ1 are lacking. Therefore, immunoprecipitation of β1was performed and the co-immunoprecipitated αvβ1 complex detected with the antibody against αv on the immunoblot. All cell lines showed only a weak signal (data not shown). Integrin heterodimer expression was also assayed by immunocytometry (Figure S1). Again, high expression of αvβ3 was found exclusively in the H28 cells, while all cells expressed moderate levels of total αv and αvβ5 but not αvβ6 or αvβ8. Finally, the high αvβ3 expression in H28 cells was confirmed by immunofluorescence (Figure 1C). Findings from these expression studies are summarised in Table 1.

**Table 1:** Summary of results in MPM: cilengitide treatment and target gene knockdown.

cell	cilengitide target expression			growth inhibition	anoikis		cilengitide-suppressed invasion		siRNA-suppressed 3D invasion	
	αv	αvβ3	αvβ5	cytotoxicity^1^	resistance^2^	death^3^	2D	3D	*ITGB3*	*ITGB5*
H28	++++	+++++	++++	+++	+	+++	+++	+++	+++	++
MM05	+++	+/−	+++	+++	+	+++	−	−	N/A	−
H226	++	+/−	+++	++	+	++	−	−	N/A	+/−
H2052	+	+/−	++	++	++	+	−	−	N/A	−
H2452	+	+/−	++	++	++	+	−	−	N/A	−
MeT-5A	+	+/−	++	++	++	++/+++	−	+/−	N/A	+/−
MSTO-211H	+	+/−	+/−	+	+++	+	−	−	N/A	−
REN	++	+/−	++	+	+++	+	+/−	+/−	N/A	−

1 Cytotoxicity (see ): +++, highly sensitive; ++, intermediate; +, relatively resistant.

2 Anoikis resistance indicated by relative % viable cells in detached culture to attached culture (see Figure S3B): +++, high resistance; ++, intermediate; +, low resistance.

3 Anoikis death in non-adherent culture (see Figure S3C): +++, high; ++, intermediate; + low.

### Cilengitide induces detachment of MPM monolayer cultures

**Figure 2:**
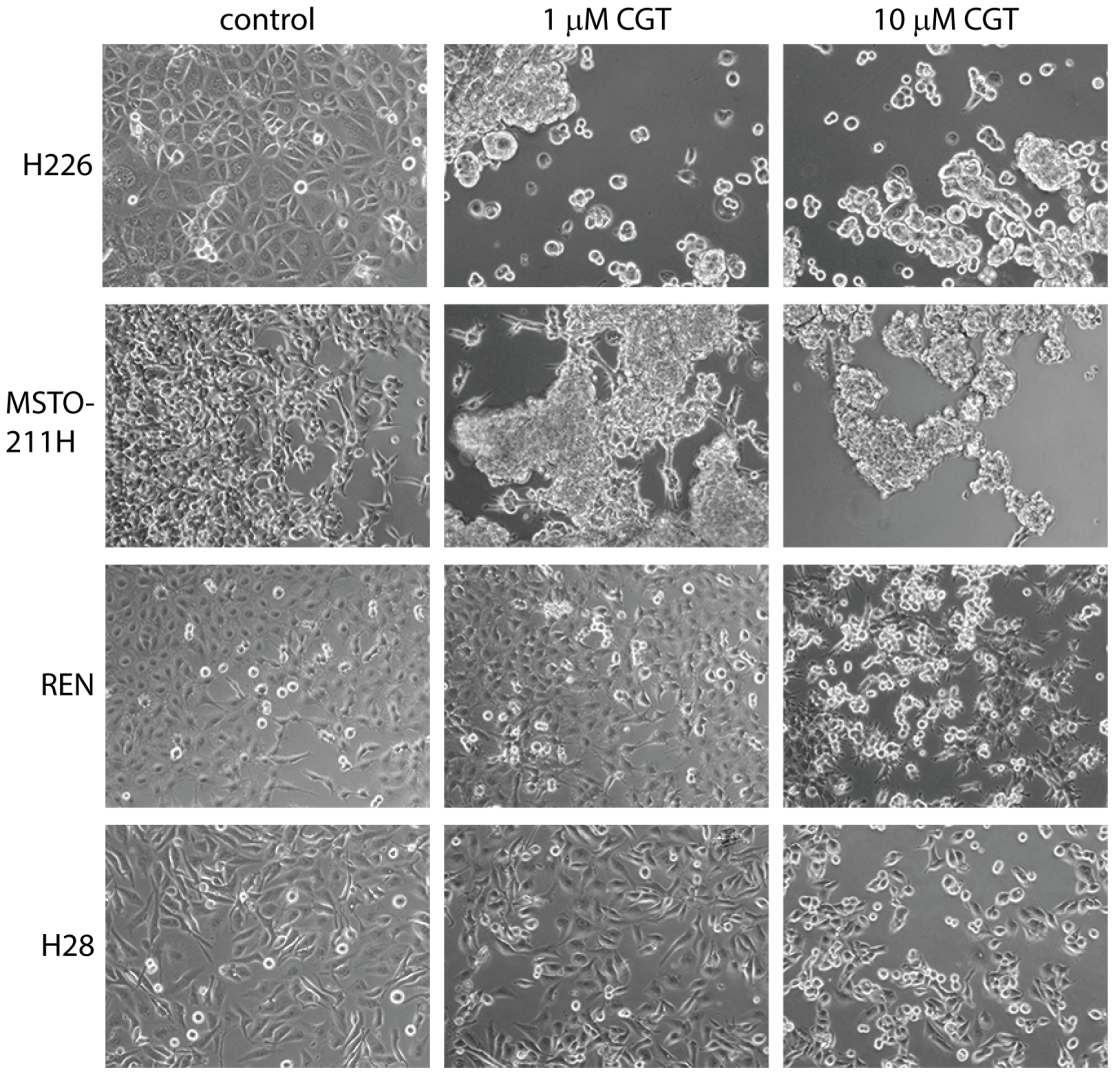
Cilengitide causes detachment of MPM cells in monolayer culture. Cells were allowed to adhere to tissue culture surface before incubation with 0, 1, or 10 µM cilengitide (CGT). Images were captured with the Zeiss Axiovert inverted microscope 1 day post-cilengitide treatment.

A prominent effect of cilengitide is cellular detachment of cells cultured on plastic surfaces [14], [28]. Indeed, cilengitide had significant effects on the adhesion and morphology of all the MPM lines in our panel. In some cases (e.g. H226 and MSTO-211H) treatment with 1 µM cilengitide for 24 h was sufficient to cause most cells to round up and detach (Figure 2), whereas in other lines (e.g. REN and H28) significant effects required higher doses of cilengitide (10 µM, Figure 2) or longer exposure (data not shown). Results for the other cell lines are shown in Figure S2.

### Cilengitide inhibits growth of MPM cells susceptible to anoikis

**Figure 3:**
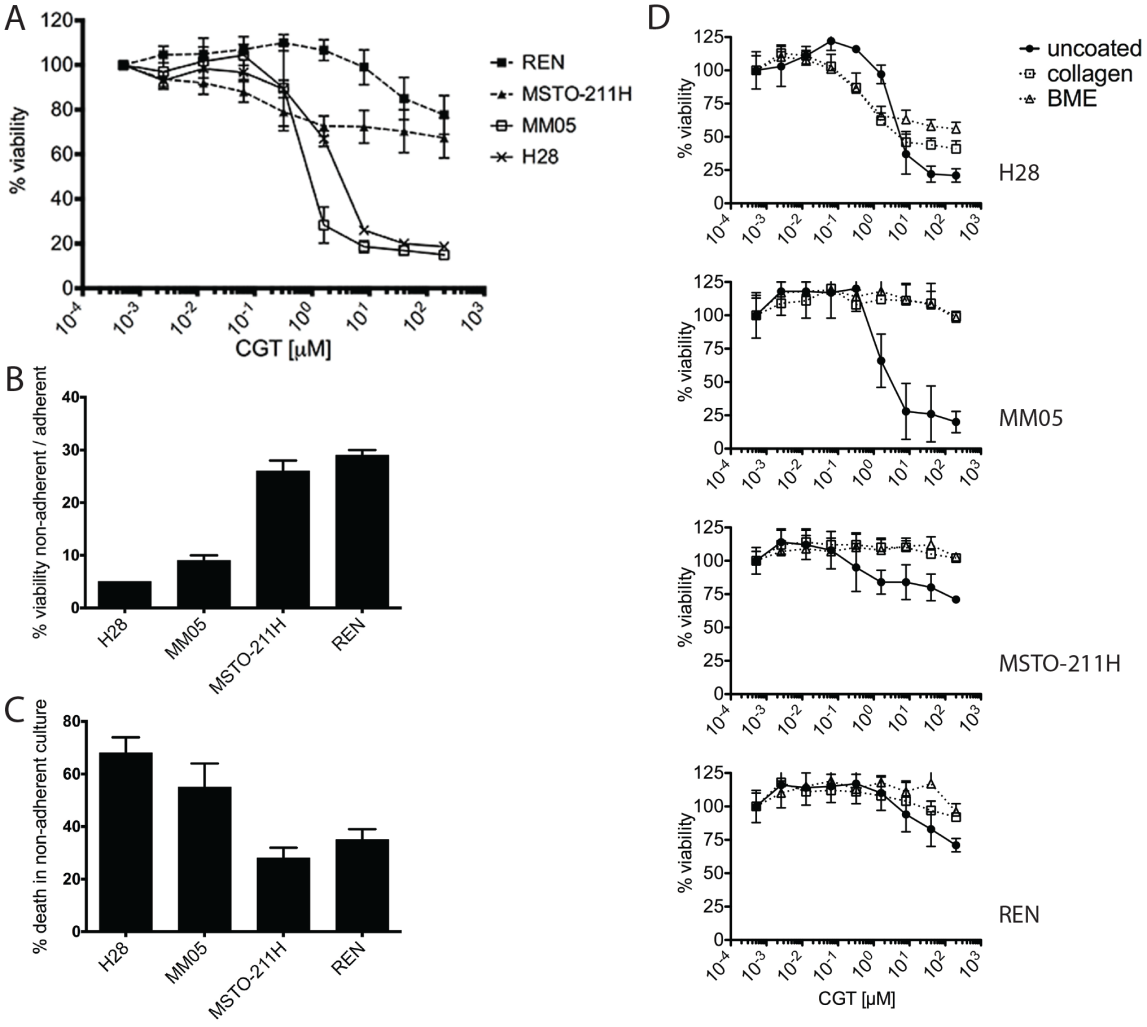
Effect of cilengitide on MPM cell viability and anchorage-independent growth. (A) Growth inhibition: cells were incubated in a concentration series of cilengitide (CGT) for 3 days. Viability was determined with the alamar blue metabolic assay. Results shown are the mean ± SD from 3 independent experiments. For simplicity, results of the 2 most sensitive and 2 most resistant cell lines were shown. (B) Anoikis resistance: cells were grown for 3 days on both ultra-low attachment plates (non-adherent condition) or on normal tissue culture plates (adherent) and viability was determined with the alamar blue metabolic assay. Anoikis-resistance is expressed as relative percentage of viable cells in non-adherent *versus* adherent culture. (C) Anoikis sensitivity is expressed as the proportion of dead cells in the non-adherent cultures, detected by ethidium homodimer III staining and calibrated to a 100% cell death control induced by saponin treatment. (D) The effect of cilengitide on proliferation of MPM cells grown on various extracellular matrix coatings. Uncoated plates were compared to plates coated with type I collagen or basal membrane extract (BME). Cells were incubated in a concentration series of cilengitide for 3 days and cell viability determined with the alamar blue assay. Results in B, C and D are means ± SD from 3 replicates.

The effect of cilengitide on growth of MPM cell lines was assessed by proliferation inhibition assay (Figure 3A and Table 1). H28 and MM05, the two cell lines with the highest expression of cilengitide target integrins, were the most sensitive to cilengitide. In contrast, MSTO-211H cells, with low expression of cilengitide target integrins, and REN cells were quite resistant. The rest of the cell line panel showed intermediate response to cilengitide (Figure S3). The growth inhibition was dose-dependent but cilengitide failed to kill all cells even at the highest concentration (200 µM) as measured by the alamar blue metabolic assay, suggesting that this compound acts in a cytostatic rather than a cytotoxic manner.

It is well known that anchorage to the ECM promotes cell survival whereas detachment is lethal to many cells - the phenomenon of anoikis [29]. It is regarded as an important mechanism of suppression of metastasis and in recent years anoikis resistance (i.e. anochorage-independent growth) has been a field of renewed interest [30], [31]. The sensitivity of MPM cells to anoikis was assessed by comparing growth and viability under adherent *versus* non-adherent conditions, i.e. on normal tissue culture plastic *versus* ultra-low attachment hydrogel-coated plates (Figure 3B & C, Figure S3 and Table 1). The lines most resistant to anoikis, MSTO-211H and REN, were also the most resistant to cilengitide in growth inhibition assays (see Figure 3A and Table 1). Conversely, the cilengitide-sensitive H28 and MM05 cells were sensitive to anoikis in non-adherent culture. This suggests that susceptibility to anoikis is a prerequisite for cilengitide-induced growth inhibition in MPM cells: i.e. cilengitide induces cell detachment (Figure 2) but only anoikis-sensitive cells succumb.

Several studies have found that cilengitide affects cell attachment to vitronectin, but not to collagen [28], [32]. Indeed, we found that cilengitide did not detach MPM cells grown on collagen. Consistent with the proposed relationship between anoikis and cilengitide sensitivity, MC and most MPM cells grown attached on plates coated with collagen or basal membrane extract became completely resistant to cilengitide (Figure 3D and Figure S3), although H28 cells became only partially resistant. Similar results were obtained from clonogenic colony formation assays on collagen-coated plates (Figure S3).

### Cilengitide does not synergize with cisplatin, gemcitabine or pemetrexed *in vitro*

Cilengitide was reported to synergize with radiotherapy or chemotherapy in pre-clinical cancer models [15], [33], [34]. Since the cytotoxicity of cilengitide as single agent was limited in MPM cells (Figure 3A), we tested it in combination with chemotherapeutic drugs commonly used to treat MPM. However, sub-toxic doses of cilengitide combined with cisplatin, gemcitabine, or pemetrexed in growth inhibition assays for 3 days had no significant effect on the toxicity of these chemotherapeutic agents (Figure S4).

### Cilengitide inhibits invasion of MPM monolayers and three dimensional (3D) spheroid cultures

**Figure 4:**
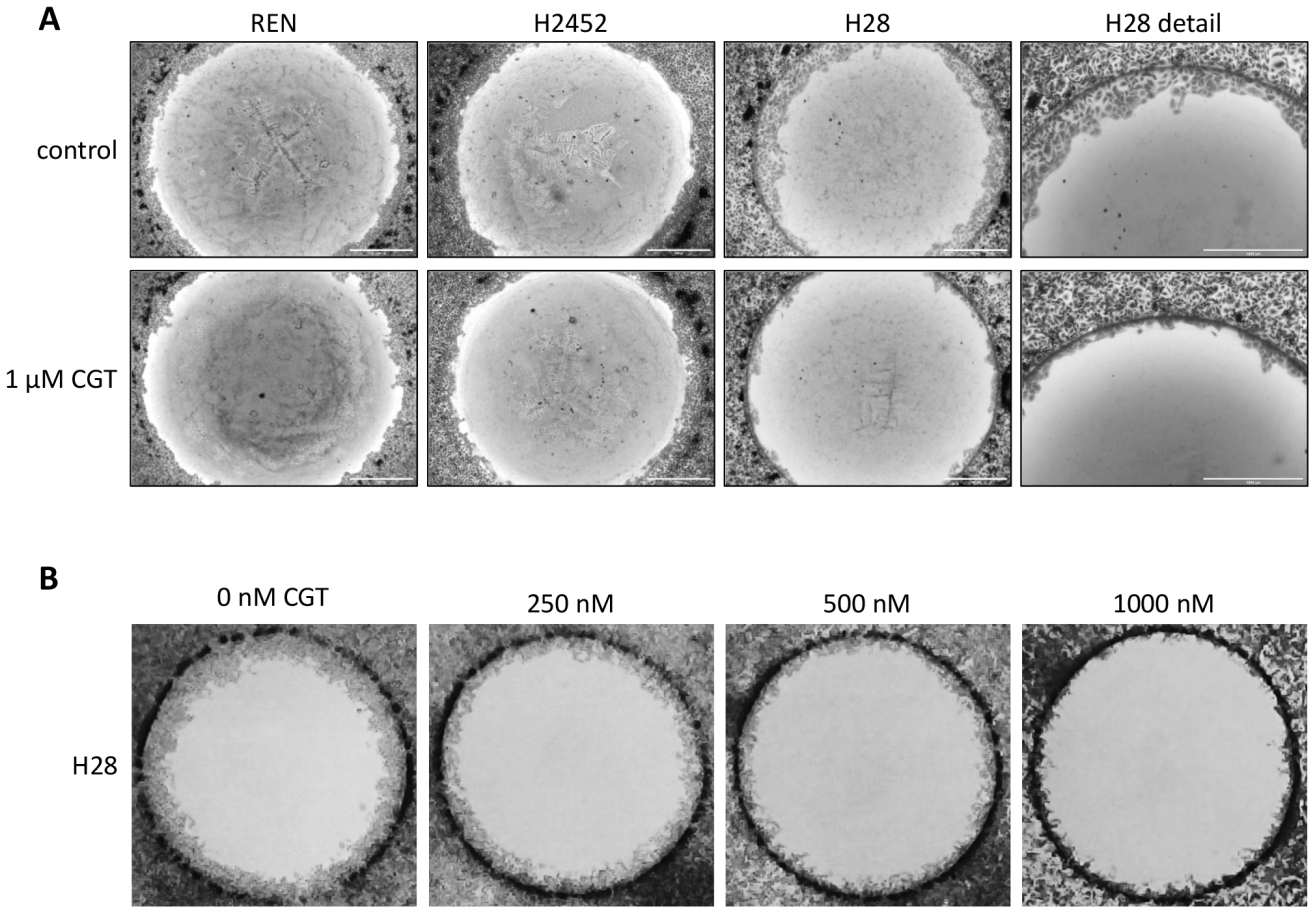
Cilengitide inhibits agarose spot invasion by H28 cells. (A) Mitotically inactivated cells were plated on wells with an agarose spot in medium ±1 µM cilengitide. After 1–2 days cells were fixed and stained with crystal violet and imaged with an EVOS-FL digital imaging system. Experiments were performed at least 3 times; representative images from three cell lines are shown. The whole agarose spots are shown in the first three columns, with the last column showing images of H28 cells at higher magnification. (B) Dose-dependent inhibition of agarose spot invasion by H28 cells. Cells were plated in medium containing various concentrations of cilengitide and imaged as above. One series is shown from 4 replicates.

Invasion is a hallmark of cancer metastasis in which integrins have a recognised role. By disrupting the interaction between integrins and their ligands, cilengitide might impact the invasiveness of MPM cells. We investigated this possibility using 2D and 3D *in vitro* models. Invasion by cells grown in monolayers was assessed using the agarose spot invasion assay, modified from Wiggins and Rappoport [35]. Cell proliferation can confound such assays so the cells were pre-treated with mitomycin C, which prevents mitosis. Cilengitide clearly suppressed invasion of H28 cells into the agarose spots (Figure 4A). The invasiveness of this cell line, with high expression of αv integrins, was reduced in a dose-dependent fashion (Figure 4B). Invasion by other cell lines however, was not substantially affected.

**Figure 5:**
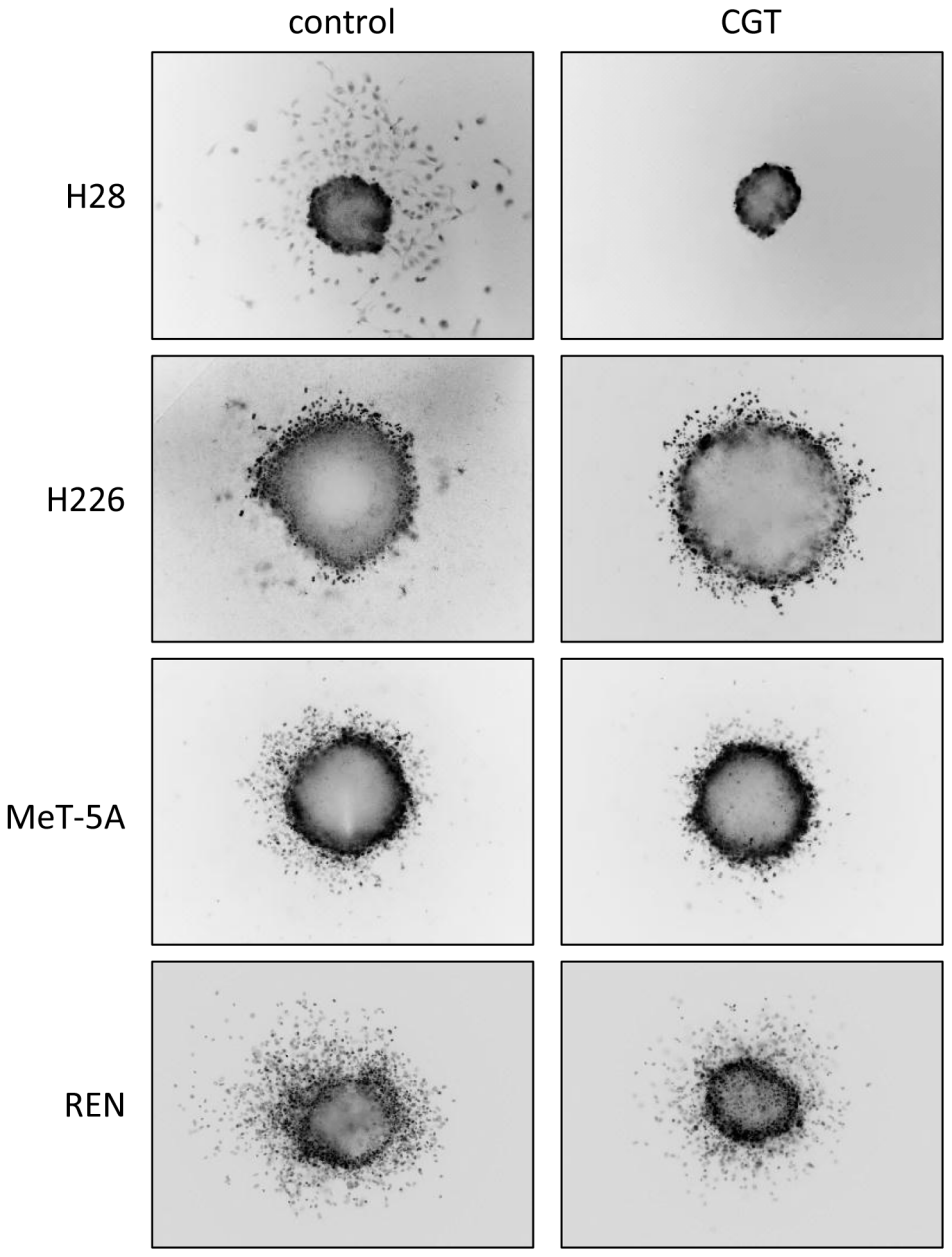
Effects of cilengitide on 3D invasion by MPM spheroids. Spheroids were embedded in collagen gel, overlaid with medium ±1 µM cilengitide and incubated at 37°C for 1–4 days. Spheroids were stained with calcein-AM or SYBR green and live images were captured. Representative images from four cell lines are shown; each experiment was performed in triplicate and on at least 3 occasions.

The invasion into collagen matrix by MPM cells from 3D tumour spheroids was also tested, which is arguably a more realistic model than the agarose spot assay. As a preliminary, we tested the effect of cilengitide on MPM spheroid growth, finding that concentrations up to 5 mM had no effect (Figure S5). Consistent with the agarose spot assay, invasion of collagen matrix by cells from H28 spheroids was completely suppressed by cilengitide (Figure 5). The behaviour of cells from spheroids of other MPM cell lines was not strongly affected (Figure 5 and Figure S6), although some inhibition of invasion was evident for the MeT-5A and REN cells. These results are also summarised in Table 1.

### Silencing *ITGB5* or *ITGB3* reduces cellular attachment and 3D invasion of MPM cells

**Figure 6:**
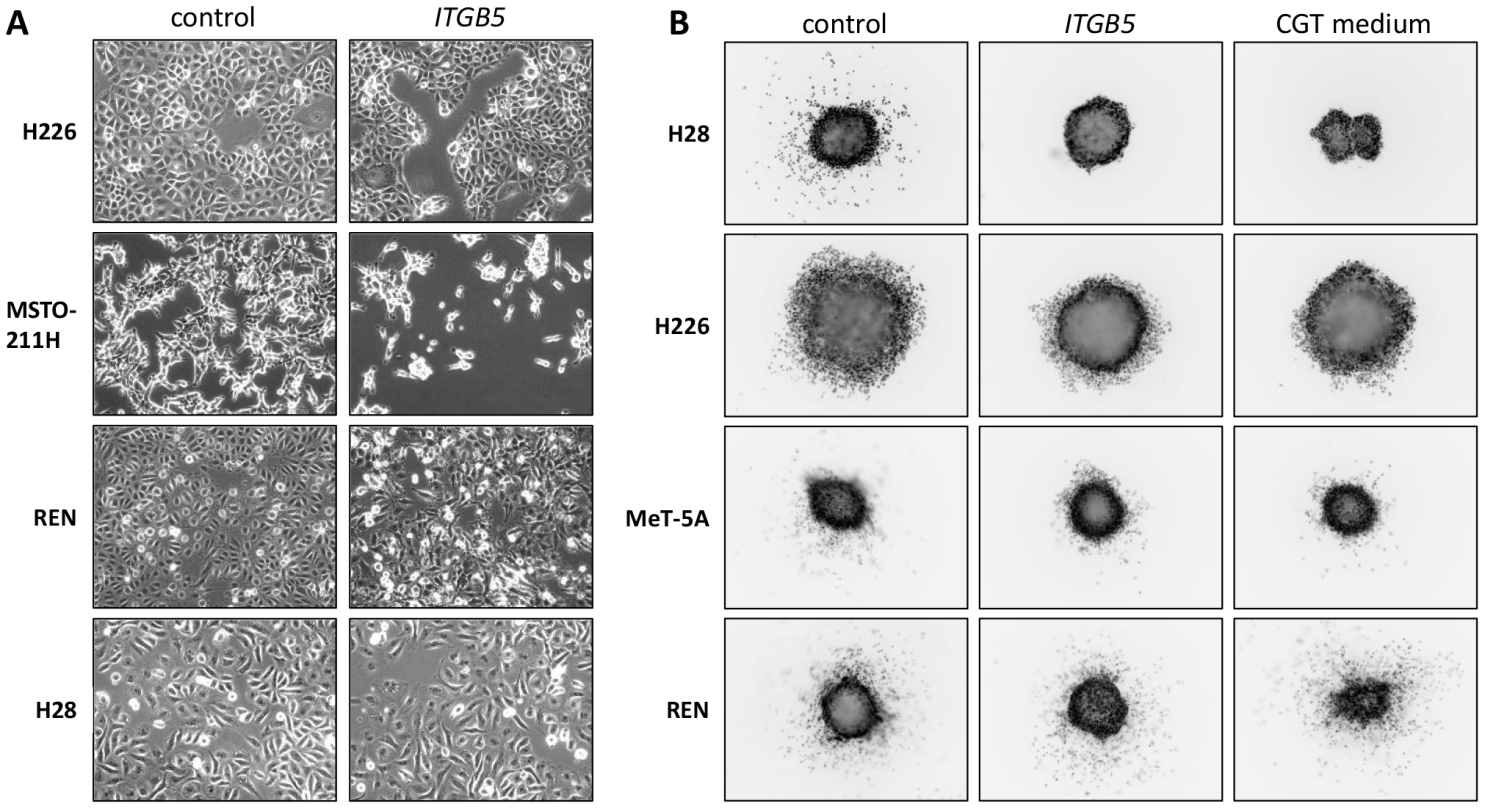
Effects of siRNA-mediated knockdown of *ITGB5* in MPM cells. (A) Morphology of MPM cells with *ITGB5* down-regulation 3 days post-siRNA transfection (compare with Figure 2). (B) 3D invasion by cells from MPM spheroids with *ITGB5* knockdown. Spheroids were generated starting one day after siRNA transfection and cultured for 3 days. They were subsequently embedded in collagen and incubated at 37°C for 1–4 days, followed by live staining with SYBR green and imaged. Representative images for 4 cell lines are shown; each experiment was performed on two occasions with 3 replicates each.

Given the effect of cilengitide on attachment and invasiveness of the MPM cells, we investigated the role of the cilengitide target integrins αvβ3 and αvβ5 in these processes by siRNA-mediated knockdowns. As shown above, αvβ3 expression was restricted to H28 cells, whereas αvβ5 was expressed moderately in all the MPM lines except MSTO-211H. Silencing the integrin β5 gene *ITGB5* with 1 nM siRNA resulted in substantial down-regulation of αvβ5 expression (Figure S7A). This did not affect MC and MPM cell growth (Figure S7B). The effects of the knockdown resembled cilengitide treatment in that most cell lines showed a more rounded morphology and increased cell detachment (Figure 6A). Moreover, as observed with cilengitide treatment, *ITGB5* knockdown suppressed invasion into collagen matrix by cells from H28 spheroids (Figure 6B). Less pronounced suppression of invasion was observed for H226 and MeT-5A spheroids.

**Figure 7:**
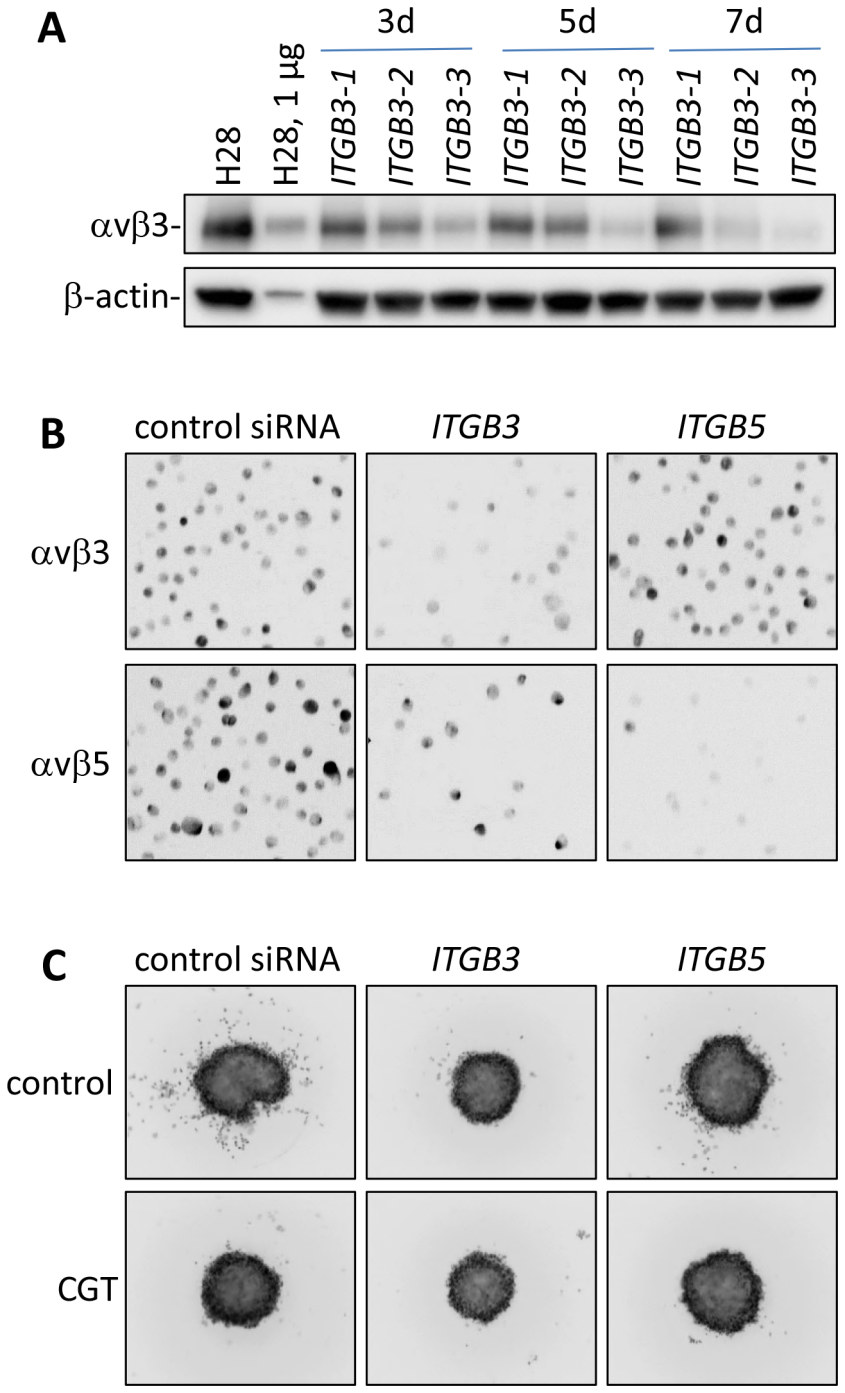
siRNA-mediated knockdown of integrin *ITGB3* and *ITGB5* in H28 cells. (A) Western analysis of αvβ3 expression 3–7 days after siRNA transfection. Three siRNAs *ITGB3-1*, *ITGB3-2* and *ITGB3*-3 were compared. Ten µg of lysates were loaded on gel except lane 2. The β-actin protein serves as loading control. (B) Immunocytometry of αvβ3 and αvβ5 in H28 cells transfected for 3 days with siRNA for *ITGB3* or *ITGB5*. Cells were immunostained with antibodies against αvβ3 or αvβ5, detected with an Alexa Fluor 488-conjugated secondary antibody and analysed with the TALI image-based cytometer. Representative fields of images captured on the cytometer are shown. (C) Invasion of collagen matrix by H28 spheroids with *ITGB3* or *ITGB5* knockdown. Spheroids were generated one day after siRNA transfection and assayed for invasion into collagen gel; each experiment was performed in triplicate and on at least 3 occasions.

The H28 cells, which showed the greatest response to cilengitide, were the only ones to express high levels of αvβ3, so it was pertinent to test the effect of *ITGB3* silencing in these cells compared to *ITGB5* (Figure 7A and 7B). Knocking down *ITGB3* with 1 nM siRNA did not affect H28 cell proliferation (not shown). However, invasion of collagen matrix by H28 spheroid cells was suppressed and to a greater extent than with *ITGB5* knockdown. Indeed, the effects were similar in extent to cilengitide treatment (Figure 7C). The suppression of MPM cell invasiveness by cilengitide is thus likely mediated by interference with the function of both αvβ5 and, when it is expressed, αvβ3. These results are also summarised in Table 1.

## Discussion

Integrins are involved in progression and angiogenesis of a variety of tumours and are potential targets for therapeutic intervention [8], [19]. Cilengitide, specifically inhibiting integrins αvβ3 and αvβ5, has shown effectiveness in a number of preclinical cancer models [15], [33], [34]. Early clinical trials showed that this drug is relatively safe at high dose [36], [37]. To investigate the potential of cilengitide in the context of malignant pleural mesothelioma, we examined its effects on mesothelial cells and a panel of MPM cell lines. A subset of the cell lines were indeed sensitive to cilengitide-induced growth inhibition in the low micromolar range, a concentration that can be achieved in human tumours [17], [37]. As seen in studies with other tumour types [15], [28], our results confirmed that cilengitide also promoted detachment of MPM cells.

Interestingly, growth inhibition induced by cilengitide did not directly correlate with cell detachment, which is a combination of several factors, but rather with higher expression of the target integrins (αvβ3 and αvβ5) and with susceptibility to anoikis i.e. cell death caused by detachment. *In vivo*, adherent cells that lose contact with extracellular matrix and become detached usually undergo death by anoikis. However, certain tumour cells can escape from anoikis and survive detachment during metastasis [30]. Cilengitide was also shown to induce cell detachment rather than direct cytotoxicity in a study of pediatric glioma cell lines, where it did not inhibit growth of normally adherent cells that were cultured under non-adherent conditions [38]. Similarly, we observed that cilengitide did not affect anchorage-independent MPM colony formation in soft agar (data not shown). Moreover, we found that the inhibition in MPM cell growth by cilengitide can be overcome by culturing them on plates coated with ECM proteins, to which they continue to adhere. Thus, the mode of action of cilengitide on MPM cells was primarily promoting detachment – only cells prone to anoikis subsequently died. However, it should be noted that adherence to ECM-coated plates only partially protected the H28 cells from cilengitide; it remains unclear whether this is merely a difference of degree or whether cilengitide has additional anti-proliferative effects on these cells.

In contrast to reports showing synergy of cilengitide with radio- or chemotherapy in other cancer models [15], [33], [34], we were unable to show synergy with cisplatin, gemcitabine and pemetrexed – the chemotherapeutic drugs used in the treatment of MPM. We speculate that this might reflect the generally poor response of MPM to chemotherapeutic agents.

Local invasion is one of the characteristics of MPM. We observed that cilengitide inhibited the invasiveness of several MPM lines in 2D and 3D models, most prominently in the H28 cells with high expression of cilengitide target genes, particularly αvβ3. Many cancers have aberrant αvβ3 expression, which is known to be associated with invasion and metastasis [19]. Yet, αvβ3 expression in MPM specimens and cell lines is infrequent [21]–[23] and indeed, we found αvβ3 expression on only one of seven MPM cell lines (H28). Nevertheless, αvβ3 may have a specific temporal role in the development of mesothelioma, which typically begins with asbestos-induced pleural inflammation. During this inflammatory process, activated, hyperplastic mesothelial cells are shed in pleural effusion. Expression of integrin αvβ3 was found to be induced in activated detached mesothelial cells and short term cultures but not in sessile mesothelial cells [39], suggesting there is temporary expression during cell detachment that might not be detected in other stages.

Another prominent effect of cilengitide seen in the αvβ3 over-expressing H28 cells and, to a lesser extent in other MPM lines, was suppression of invasion. We have, in addition, analysed primary mesothelioma cultures and observed suppression of 3D invasion by cilengitide in one sample out of six, which did express αvβ3 (results not shown). Integrin αvβ3 expression is more common in glioblastoma and Maurer *et al.* [28] have shown that invasion in the transwell assay by two out of three glioblastoma cell lines with high αvβ3 expression was suppressed by cilengitide. We have confirmed this result in the H28 cells with the transwell system (not shown).

The role of αvβ3 and αvβ5 in MPM responses to cilengitide was confirmed by gene knockdown of the respective beta subunit genes *ITGB3* and *ITGB5*. The knockdowns caused cellular detachment and suppressed invasion of the matrix by spheroid cells and mimicked the effect of cilengitide.

The effects of cilengitide were most pronounced in H28 cells with high αvβ3 expression, including growth inhibition. Correlations between growth inhibition by cilengitide and αvβ3 expression level were also reported for neuroblastoma and pediatric glioma cells [38]. The *ITGB3* and *ITGB5* knockdowns in the MPM cells were deliberately performed with a low, non-toxic concentration of siRNA (1 nM) to exclude non-specific toxicities of the siRNA oligonucleotide. At higher concentrations of siRNA growth inhibition did occur (results not shown) but it is not clear whether this is related to silencing of *ITGB3* or *ITGB5*.

In summary, the expression of integrins targeted by cilengitide likely contributes to the invasiveness of MPM. Cilengitide is able to suppress the growth of MPM cells by promoting their detachment, provided they are prone to anoikis. Moreover, in 3D models cilengitide is able to suppress the invasiveness of MPM cells that express high levels of its targets. These effects evidently arise from antagonism of integrins αvβ3 and αvβ5, as they are reproduced by silencing of the respective integrin beta subunit genes. These results bolster the argument that integrins should be considered as potential targets for the treatment of MPM.

## Materials and Methods

### Cells

The MPM cell lines H28, H226, H2052, H2452, MSTO-211H and the mesothelial cell line MeT-5A were purchased from the American Type Culture Collection (ATCC). MM05 cells [40] were established by Kwun Fong's laboratory (The Prince Charles Hospital, Brisbane, Australia). REN cells [41] were obtained from Laura Moro who originally received the cells from Steven Albelda (University of Pennsylvania, Phildelphia, USA). All cells were grown in RPMI 1640 supplemented with 10% fetal bovine serum (FBS, Life Technologies, Mulgrave, Victoria, Australia) hereafter referred to as complete medium.

### Quantitative real time RT-PCR

Total RNA was isolated with the guanidinium thiocyanate-phenol-chloroform extraction method using TRIzol reagent (Life Technologies). Reverse transcription was performed on 1 µg RNA in 20 µl volume with the AffinityScript qPCR cDNA synthesis kit according to the manufacturer's directions (Agilent Technologies, Santa Clara, California, USA). The cDNA was diluted with nuclease-free water and real-time qPCR was performed on 5 ng equivalent of starting RNA with the Brilliant II SYBR green qPCR master mix (Agilent Technologies) on a Stratagene Mx3000P cycler with an initial incubation at 95°C for 10 minutes, followed by 40 cycles of 95°C for 15 seconds and 55°C for 30 seconds, and a final cycle of 95°C for 1 minute, 55°C for 30 seconds, and 95°C for 30 seconds and analysed with the MxPro qPCR software. Expression data were normalised to the glyceraldehyde-3-phosphate dehydrogenase (*GAPDH*) gene. See Table S1 for primer sequences.

### Western blot analysis and co-immunoprecipitation

Cell lysates were resolved by SDS-PAGE under non-reducing conditions, blotted onto PVDF or nitrocellulose membranes (Bio-Rad, Hercules, California, USA), and probed with rabbit monoclonal antibodies against integrins αv, αvβ3, αvβ5, αvβ6, or αvβ8 ([27], kindly provided by Merck KGaA, Darmstadt, Germany). The antigens were detected using a peroxidase-conjugated secondary antibody (Thermo Fisher Scientific, Waltham, Massachusetts, USA) and SuperSignal WestFemto ECL reagent (Thermo Fisher Scientific). Chemiluminescence signals were captured with a GEL LOGIC 2200 imaging system and analysed with the Kodak MI SE software. For the detection of the αvβ1 complex immunoprecipitation was performed with a β1 antibody (Millipore clone P4C10, Merck KGaA). The immune complexes were eluted in non-reducing sample buffer, resolved by SDS-PAGE, immunoblotted and detected with the rabbit monoclonal antibody against αv.

### Image-based immunocytometry

Cells were detached with trypsin-EDTA (Life Technologies), fixed with 3.7% paraformaldehyde (Sigma-Aldrich, St. Louis, Missouri, USA), and stained with antibodies against αv, αvβ3, αvβ5, αvβ6, or αvβ8 [27], followed by incubation with an Alexa Fluor 488-conjugated secondary antibody (Life Technologies). Cell suspension was analysed with a TALI image-based cytometer (Life Technologies). Nine machine-selected fields on the slide were screened and relative fluorescence per cell was calculated after subtraction of background fluorescence. The images captured on the cytometer were processed with Image J software.

### Immunofluorescence

Cells were allowed to adhere overnight on chamber slides (Thermo Fisher Scientific) before fixation with 3.7% paraformaldehyde. Subsequently, cells were permeabilised with 0.1% Triton in PBS, blocked with 10% FBS in PBS, incubated with the antibody against αvβ3 and detected with an Alexa Fluor 488-conjugated secondary antibody. Immunostaining was visualised with a Zeiss Axiovert inverted fluorescence microscope using the FITC filter and AxioVision software. Images were further processed with Image J software.

### Cell proliferation assay

Cells were dispensed in complete medium to 96-well plates (3000 cells/well) and allowed to adhere overnight prior to addition of cilengitide (kindly provided by Merck KGaA) for 72 h. Plates were either uncoated tissue culture grade plates, or coated with collagen type I (Life Technologies) or basal membrane extract (BME) purified from murine Engelbreth-Holm-Swarm tumour (Geltrex, Life Technologies) to enhance cell attachment. Cell viability was then quantified with the alamar blue metabolic assay using the FluoStar OPTIMA plate reader (BMG LABTECH, Ortenberg, Germany, excitation 530 nm, emission 590 nm). To test for synergy of cilengitide with chemotherapeutic drugs, sub-toxic concentrations of cilengitide (0, 0.1, 0.2, 0.4 or 1 µM) were combined with a concentration series of the cytotoxic drugs cisplatin (Pfizer, New York, NY, USA), gemcitabine (Actavis, Dublin, Ireland) or pemetrexed (Eli Lilly, Indianapolis, Indiana, USA) and incubated and analysed as above.

### Cell attachment and non-adherent culture

For cell attachment assays, cells were dispensed in complete medium into 6-well plates at 50% confluency. The next day the cultures were replenished with medium containing various concentrations of cilengitide, each in duplicate wells. The plates were examined daily for changes in cellular morphology and attachment. Images were captured with a Zeiss Axiovert inverted microscope and AxioVision software. For anoikis studies, cells were plated (3000 cells/well) onto 96-well tissue culture plates (adherent culture) or ultra-low attachment (Sigma-Aldrich) plates (non-adherent culture) for 3 days. Cell viability was quantified with alamar blue on a FluoStar OPTIMA plate reader as above. Anoikis resistance was indicated by the ratio of signal in non-adherent culture *versus* adherent culture. Anoikis death of non-adherent cultures was quantifed with ethidium homodimer III (Biotium, Hayward, California, USA) staining on the plate reader with 530/612 nm excitation and emission filters. Wells treated with 0.1% saponin (Sigma-Aldrich) represented 100% death controls.

### Clonogenic assay

Cells were seeded in complete medium at 3000/well on 6-well plates coated with type I collagen (Life Technologies). The next day, the medium was replaced with medium ±1 µM cilengitide, each in duplicate wells. Medium was refreshed every 3 to 4 days until colonies were macroscopically visible. Plates were then stained with crystal violet and imaged.

### Monolayer agarose spot invasion assay

The agarose spot invasion assay was modified from Wiggins and Rappoport [35] who showed chemotactic invasion by MDA-MB-231 cells into EGF-containing agarose spots. We have adapted this method into a universal invasion assay for most cell types. In brief, a 10 µl droplet of 1% agarose in PBS containing 0.25% serum was spotted in the centre of each well of 24-well plates. The plates were then coated with Geltrex basal membrane extract (Life Technologies) according to the manufacturer's directions. To ensure that the observed effects were not due to confounding anti-proliferative effect of cilengitide, the cells were first mitotically inactivated with 10 µg/ml mitomycin C (Sigma-Aldrich) for 2 hours. Cells were then plated at high density (∼1–2×10^5^ cells per well) in complete medium ±1 µM cilengitide and cultured for 1–3 days to assess invasion of the agarose spot. Plates were then stained with crystal violet and imaged using an EVOS-FL digital microscope (Life Technologies).

### 3D-tumour spheroid culture and collagen matrix invasion assay

A 50 µl aliquot of 1% agarose in PBS was dispensed to each well of 96 well plates to create a concave non-adherent surface. To form tumour spheroids, cells (2500 or 5000 per well depending on cell line) were plated on top of the agarose in complete medium, concentrated by gentle centrifugation at 800 rpm for 5 minutes, and incubated at 37°C for 3–4 days. Similar results were obtained with ultra-low attachment (ULA) 96-well round-bottomed plates (Sigma-Aldrich).

Tumour spheroids were gently transferred to a round-bottomed plate and embedded in 1.5% type I collagen (Life Technologies) in PBS containing 5% serum. The plate was incubated at 37°C for at least 30 min to allow collagen to set. The collagen gel was overlaid with complete medium ±1 µM cilengitide. Invasion was apparent within 1–4 days. Cells invading the collagen matrix were visualised with calcein AM or SYBR Green (Life Technologies) staining and imaged with a Zeiss Axiovert inverted fluorescence microscope and AxioVision software.

### *ITGB3* and *ITGB5* gene knockdown

The MC and MPM cells were reverse transfected overnight with 1 nM siRNA (see Table S1 for sequences) using the Lipofectamine RNAiMAX reagent (Life Technologies) according to the manufacturer's directions. Three siRNAs were tested for each gene; the one used for experiments was the most effective one that did not affect proliferation.

## Supporting Information

Figure S1 **Expression analysis of cilengitide target integrins in MPM cells by immunocytometry.** Levels of αv integrins in MPM cells were measured using image-based immunocytometry with a TALI cytometer. The mean relative fluorescence of cells in 9 fields was plotted after subtraction of background fluorescence.
(PDF)

Figure S2 **Cilengitide causes detachment of MPM cells in monolayer cultures.** Results are shown for the 4 cell lines omitted from Figure 2 in the text.
(PDF)

Figure S3 **Effect of cilengitide on MPM cell viability and anchorage-independent growth.** Figures S3A, B and C are equivalent to Figures 3A, B and C in the text but show results for all 8 cell lines or lines omitted from Figure 3 in the text. (D) Clonogenic assay. Cells were attached on collagen-coated wells and cultured in complete medium ±1 µM cilengitide and stained with crystal violet.
(PDF)

Figure S4 **Effect of cilengitide on cytotoxicity of cisplatin, gemcitabine and pemetrexed in MPM cells.** Cells were incubated in a concentration series of cytotoxic drugs ±1 µM cilengitide for 3 days. (A) cisplatin. (B) gemcitabine. (C) pemetrexed.
(PDF)

Figure S5 **Effect of cilengitide on growth of MPM spheroids versus monolayer cultures.** Spheroids and monolayer cells were incubated in a concentration series of cilengitide for 3 days and viability determined with the alamar blue assay.
(PDF)

Figure S6 **Effect of cilengitide on 3D invasion by MPM spheroids.** Results are shown for the 4 cell lines omitted from Figure 5 in the text.
(PDF)

Figure S7 **Effects of siRNA-mediated knockdown of** ***ITGB5*** **in MPM cells.** (A) Level of *ITGB5* down-regulation measured with the TALI image-based cytometer. (B) Growth curves for MPM cells after transfection with 1 nM of control or *ITGB5* siRNA. (C) 3D invasion by cells from MPM spheroids with *ITGB5* knockdown showing results of the 4 cell lines omitted from Figure 6B in the text.
(PDF)

Table S1 **qPCR primers and siRNA sequences.**
(PDF)
