# Supplementary figures and images for: Cilengitide Inhibits Attachment and Invasion of Malignant Pleural Mesothelioma Cells through Antagonism of Integrins αvβ3 and αvβ5
Source: PLoS One. 2014 Mar 3;9(3):e90374. doi: 10.1371/journal.pone.0090374 (PMC3940880; doi:10.1371/journal.pone.0090374)

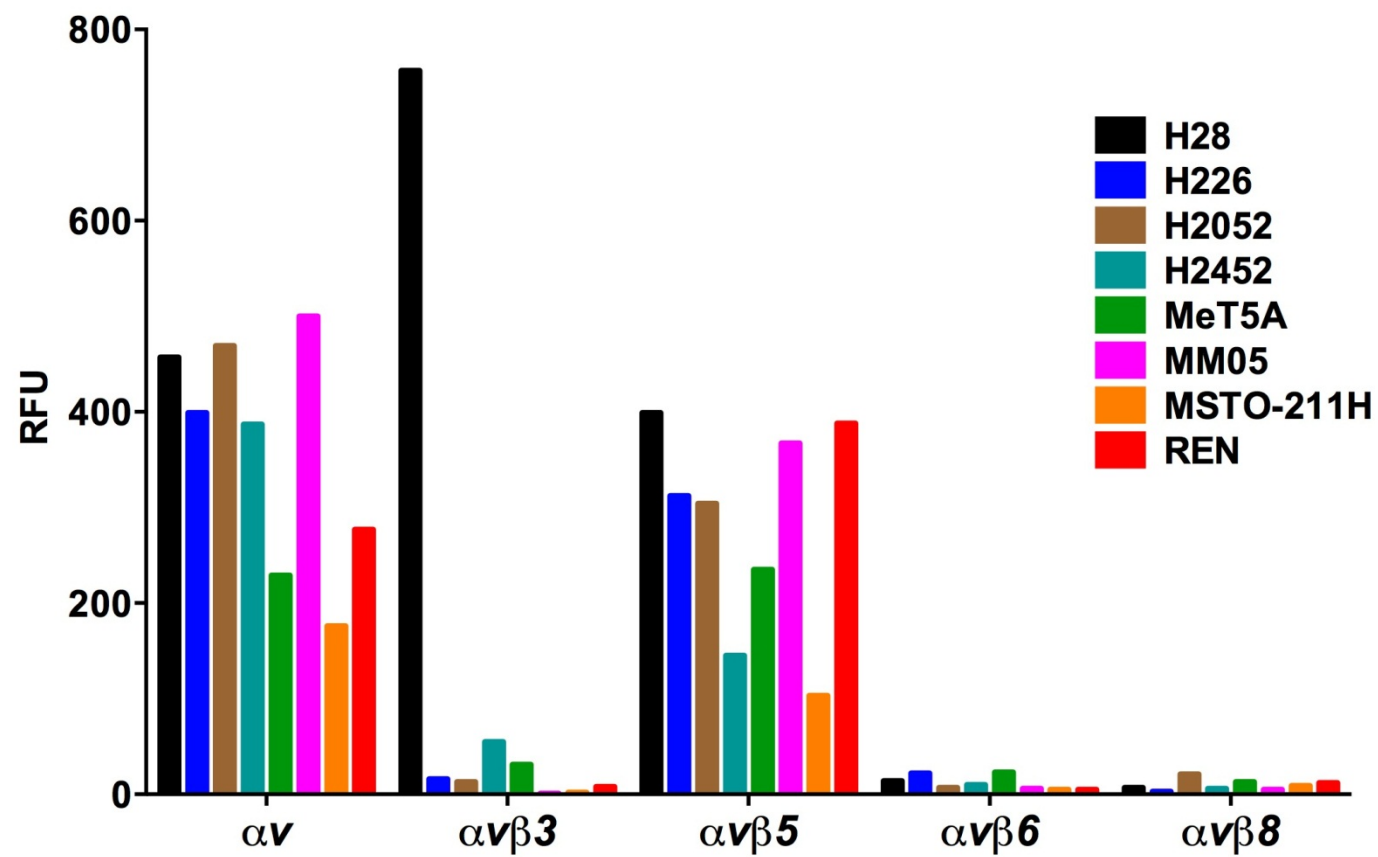

Supplement: Figure S1 — Expression analysis of cilengitide target integrins in MPM cells by immunocytometry. Levels of αv integrins in MPM cells were measured using image-based immunocytometry with a TALI cytometer. The mean relative fluorescence of cells in 9 fields was plotted after subtraction of background fluorescence. (PDF) [file pone.0090374.s001.pdf]

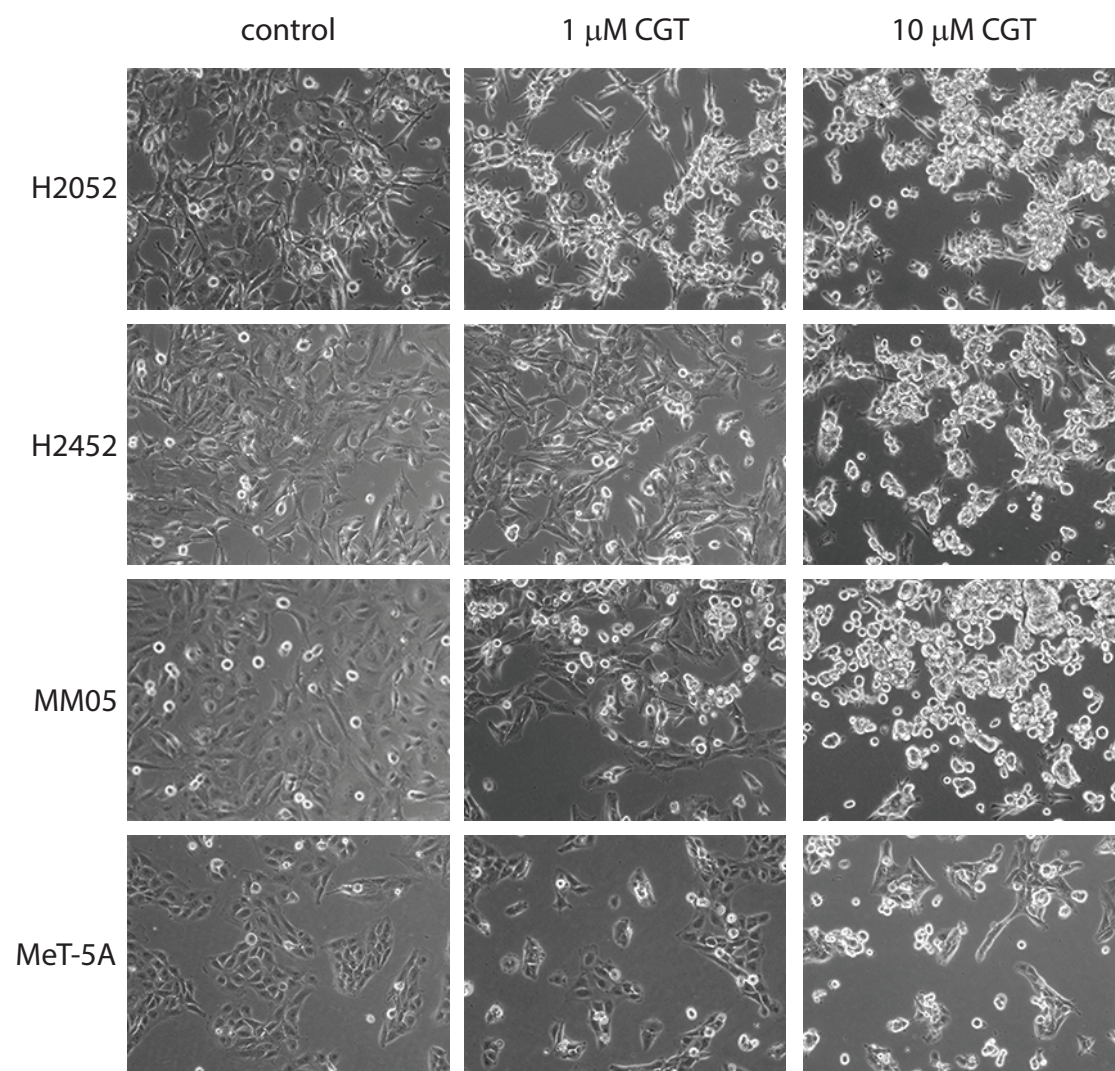

Supplement: Figure S2 — Cilengitide causes detachment of MPM cells in monolayer cultures. Results are shown for the 4 cell lines omitted from Figure 2 in the text. (PDF) [file pone.0090374.s002.pdf]

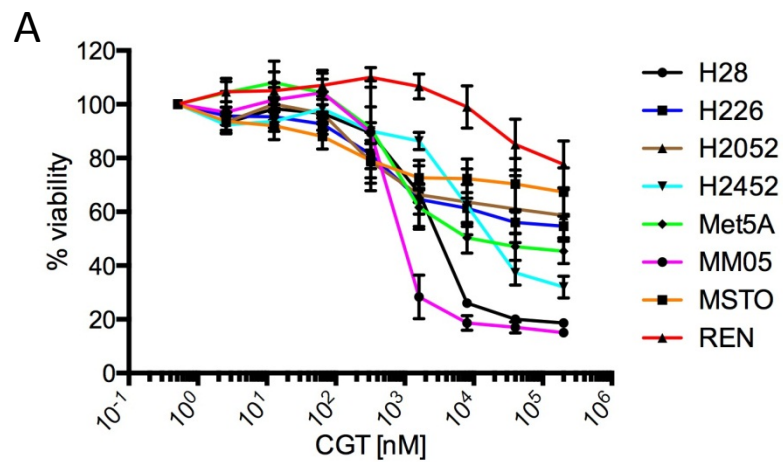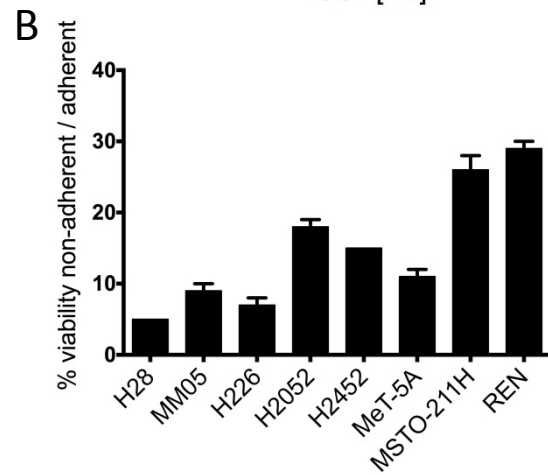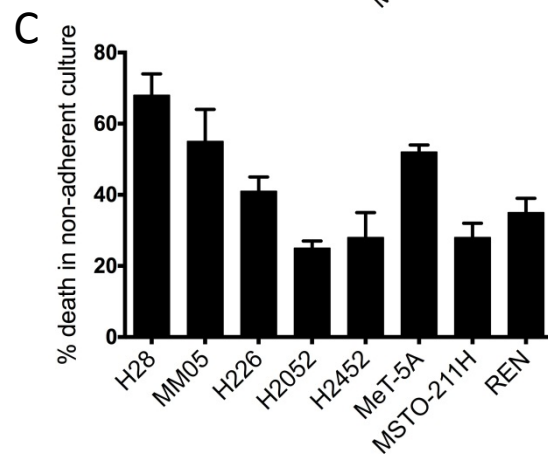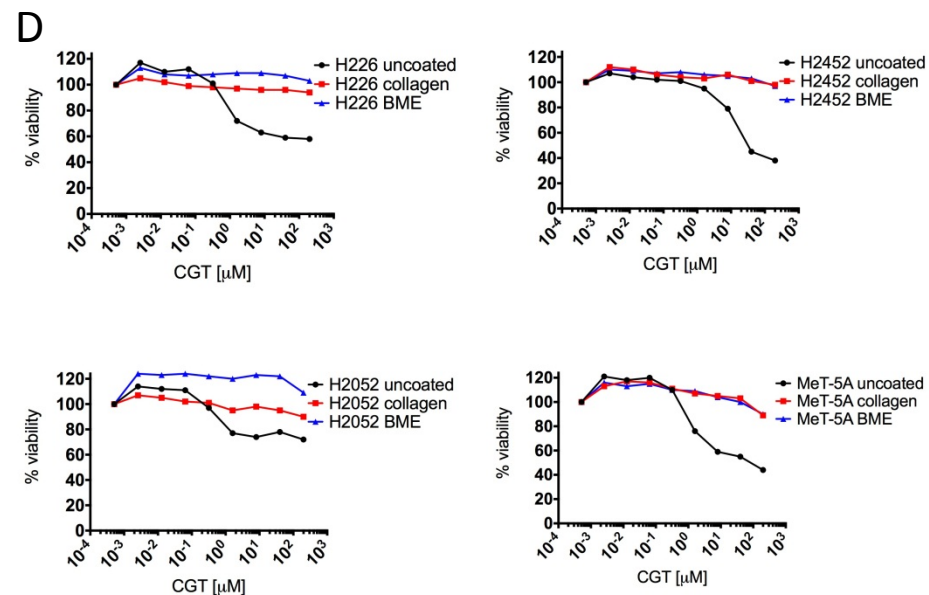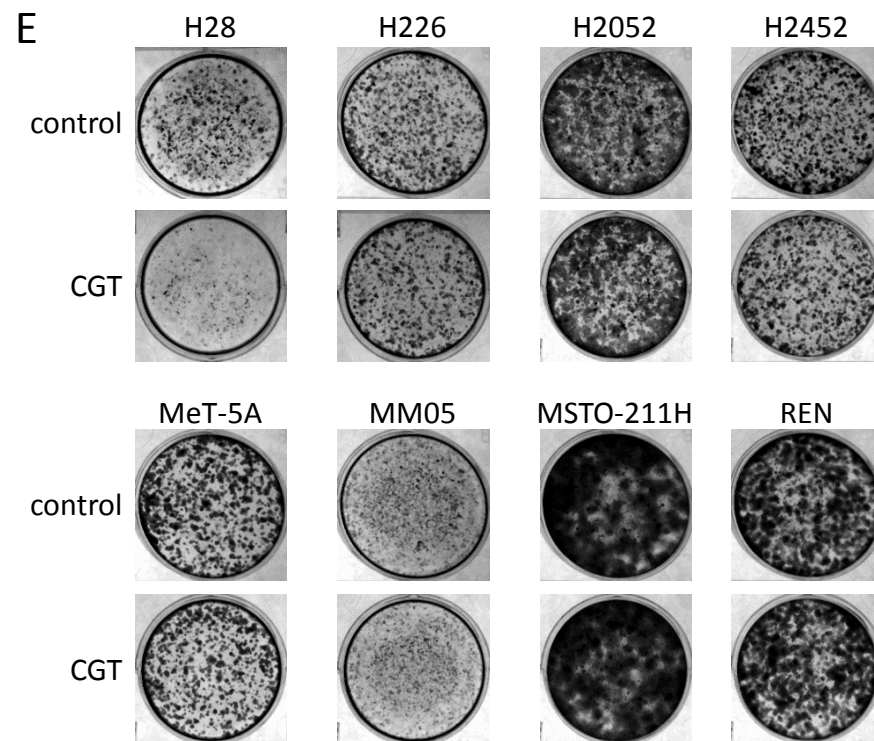

Supplement: Figure S3 — Effect of cilengitide on MPM cell viability and anchorage-independent growth. Figures S3A, B and C are equivalent to Figures 3A, B and C in the text but show results for all 8 cell lines or lines omitted from Figure 3 in the text. (D) Clonogenic assay. Cells were attached on collagen-coated wells and cultured in complete medium ±1 µM cilengitide and stained with crystal violet. (PDF) [file pone.0090374.s003.pdf]

A

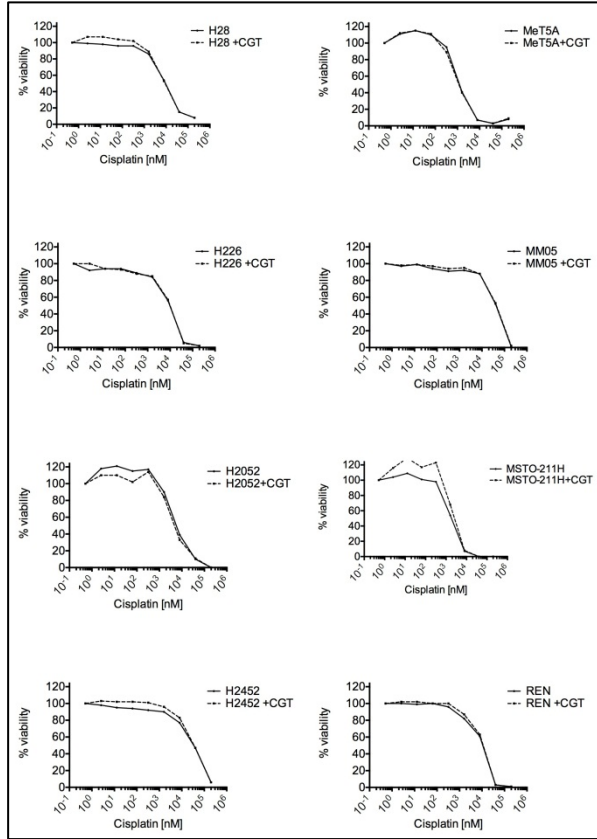

B

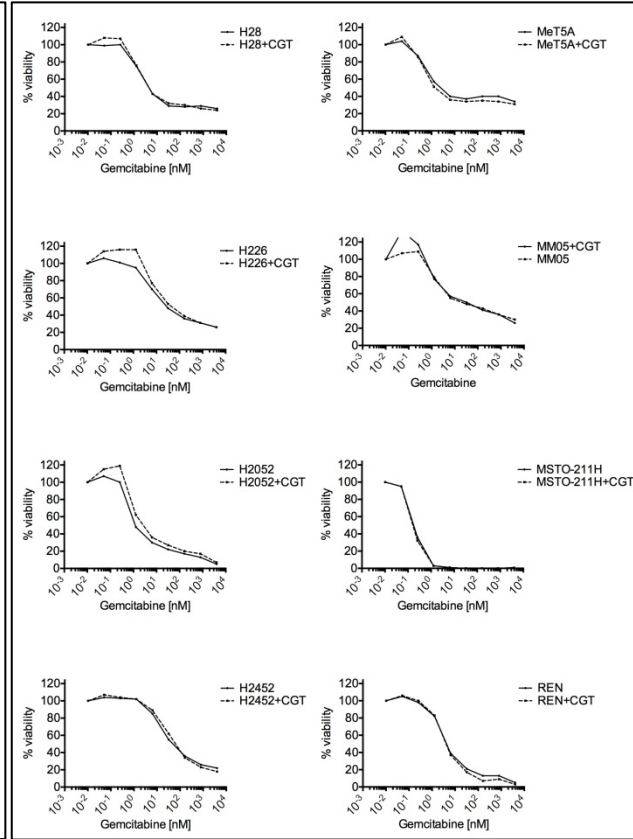

C

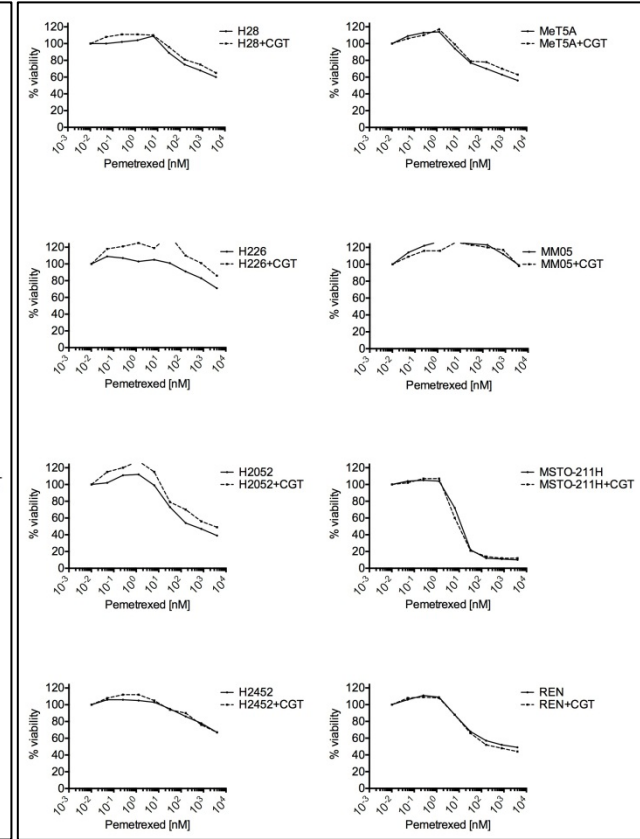

Supplement: Figure S4 — Effect of cilengitide on cytotoxicity of cisplatin, gemcitabine and pemetrexed in MPM cells. Cells were incubated in a concentration series of cytotoxic drugs ±1 µM cilengitide for 3 days. (A) cisplatin. (B) gemcitabine. (C) pemetrexed. (PDF) [file pone.0090374.s004.pdf]

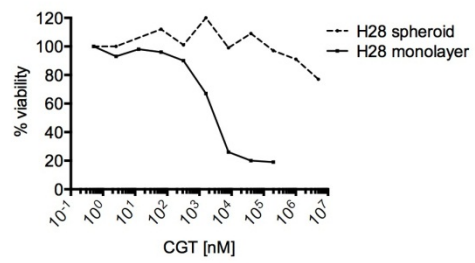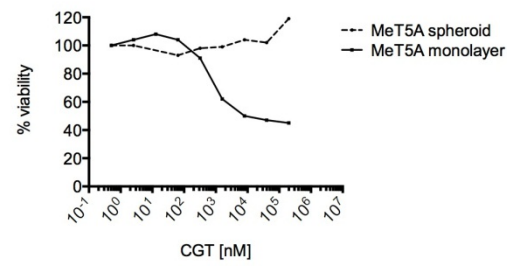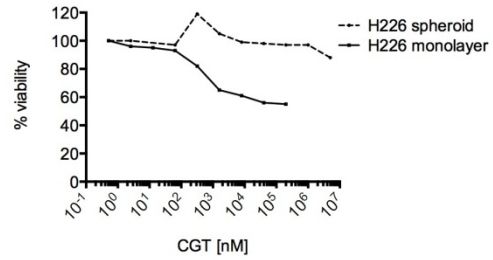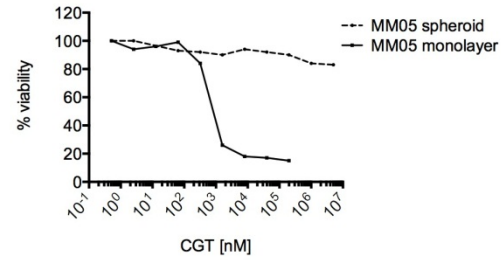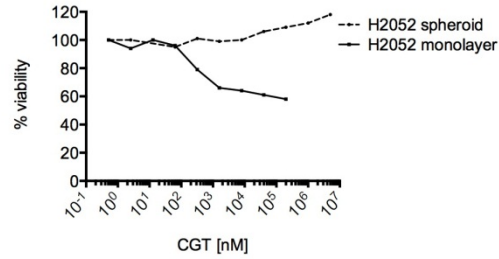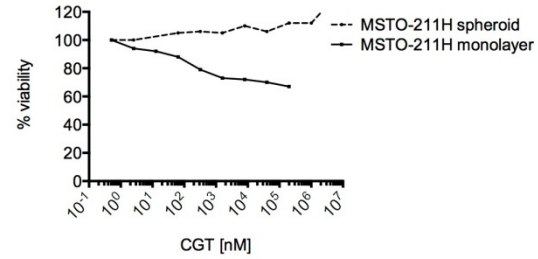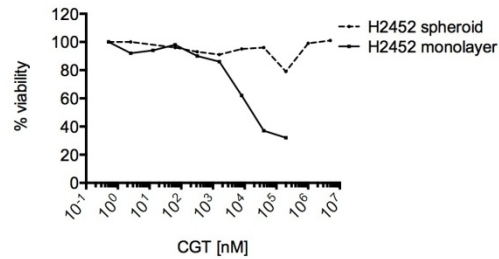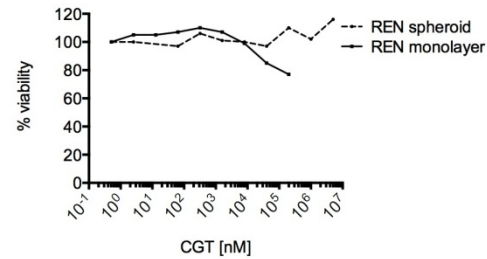

Supplement: Figure S5 — Effect of cilengitide on growth of MPM spheroids versus monolayer cultures. Spheroids and monolayer cells were incubated in a concentration series of cilengitide for 3 days and viability determined with the alamar blue assay. (PDF) [file pone.0090374.s005.pdf]

control

CGT

H2052

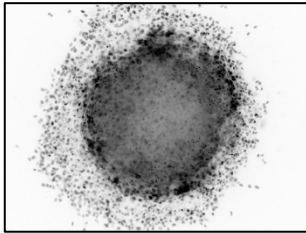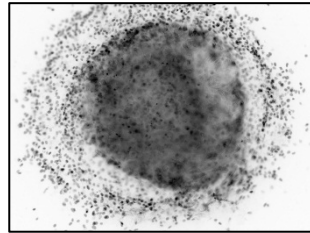

H2452

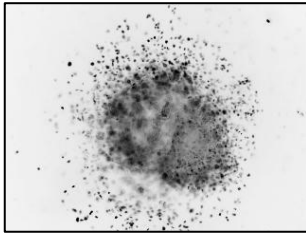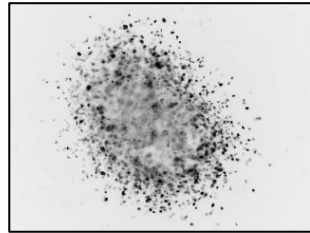

MM05

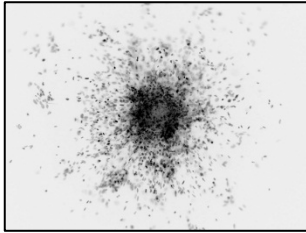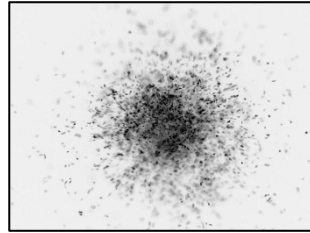

MSTO-  
211H

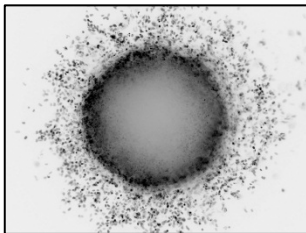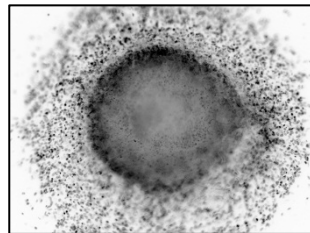

Supplement: Figure S6 — Effect of cilengitide on 3D invasion by MPM spheroids. Results are shown for the 4 cell lines omitted from Figure 5 in the text. (PDF) [file pone.0090374.s006.pdf]

A

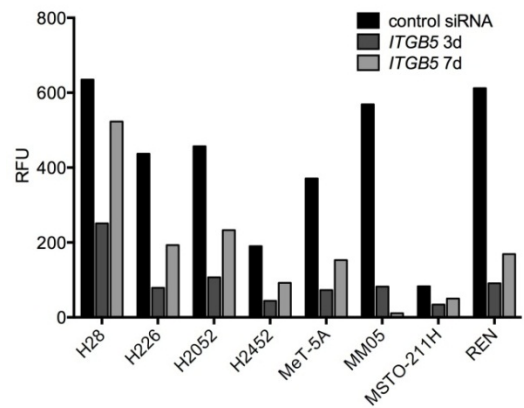

B

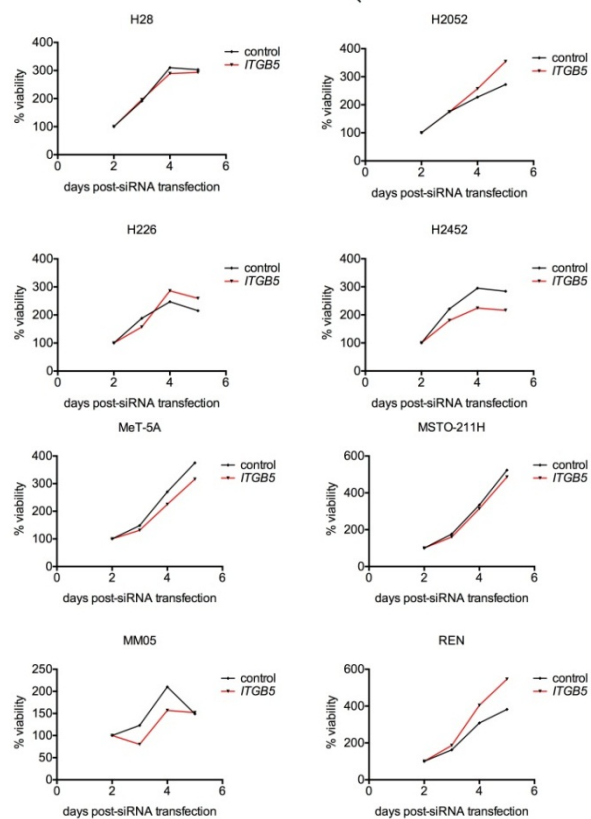

C

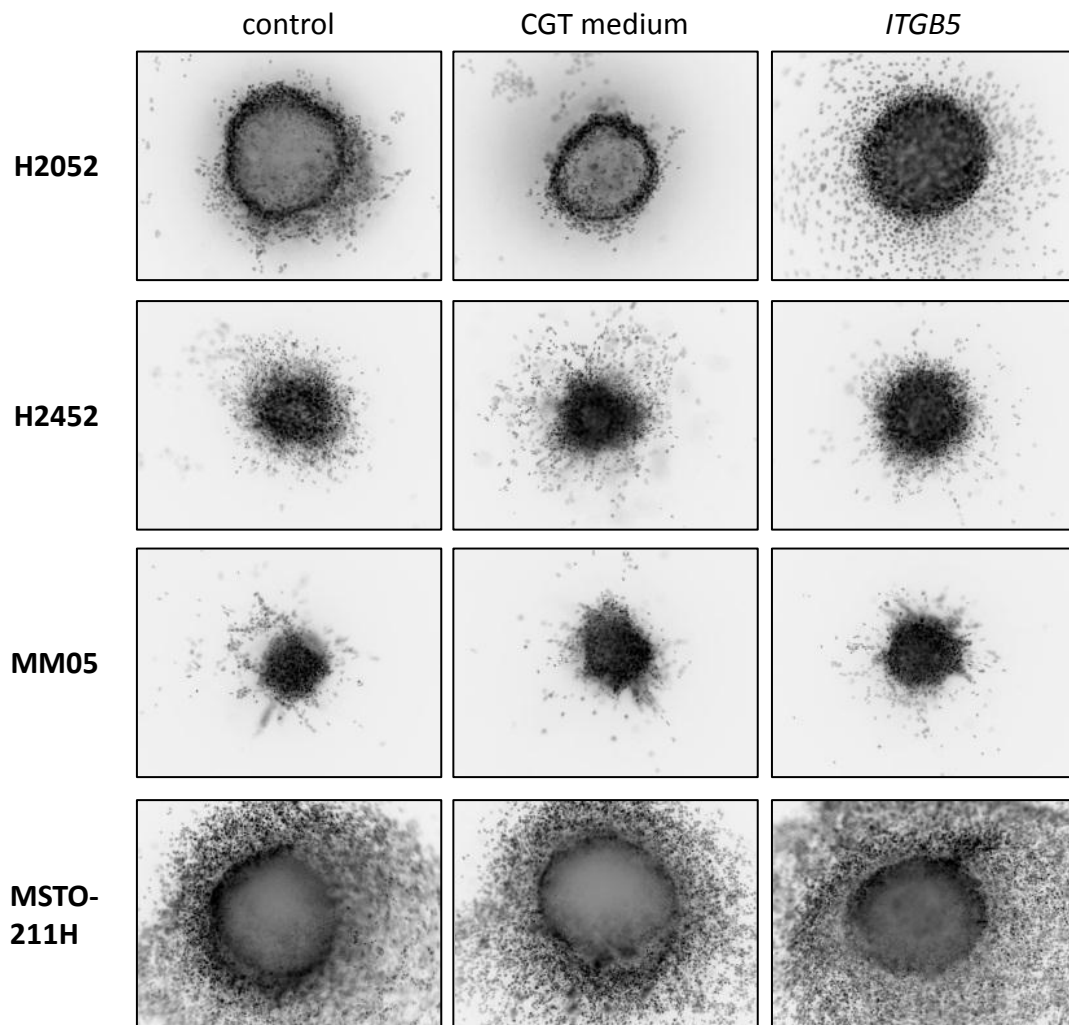

Supplement: Figure S7 — Effects of siRNA-mediated knockdown of ITGB5 in MPM cells. (A) Level of ITGB5 down-regulation measured with the TALI image-based cytometer. (B) Growth curves for MPM cells after transfection with 1 nM of control or ITGB5 siRNA. (C) 3D invasion by cells from MPM spheroids with ITGB5 knockdown showing results of the 4 cell lines omitted from Figure 6B in the text. (PDF) [file pone.0090374.s007.pdf]
