# Supplementary material for: Cilengitide Inhibits Attachment and Invasion of Malignant Pleural Mesothelioma Cells through Antagonism of Integrins αvβ3 and αvβ5
Source: PLoS One. 2014 Mar 3;9(3):e90374. doi: 10.1371/journal.pone.0090374 (PMC3940880; doi:10.1371/journal.pone.0090374)
Supplement: Table S1 — qPCR primers and siRNA sequences. (PDF) [file pone.0090374.s008.pdf]

Table S1: qPCR primers and siRNA sequences.

Real-time qPCR primers

**Genes**	**Forward primer**	**Reverse primer**
ITGAV	GCCGTGGATTTCTTCGTG	GAGGACCTGCCCTCCTTC
ITGB1	CGATGCCATCATGCAAGT	ACACCAGCAGCCGTGTAAC
ITGB3	CGCTAAATTTGAGGAAGAACG	GAAGGTAGACGTGGCCTCTTT
ITGB5	GGAGTTTGCAAAGTTTCAGAGC	TGTGCGTGGAGATAGGCTTT
ITGB6	GAAGGAATGATCACGTACAAGGT	AGCAGGCAGTCTTCACAGGT
ITGB8	GGCCAAGGTGAAGACAATAGA	ATCCTCTTGAACACACCATCC

siRNA sequences

**siRNA**	**Sense**	**antisense**
ITGB3-siRNA1	GCUUCAAUGAGGAAGUGAATT	UUCACUUCCUCAUUGAAGCTT
ITGB3-siRNA2	GCUCAUUGUUGAUGCUUAUTT	AUAAGCAUCAACAAUGAGCTT
ITGB3-siRNA3	GCAGUGAAUUGUACCUAUATT	UAUAGGUACAAUUCACUGCTT
ITGB5-siRNA1	GCUCGCAGGUCUCAACAUATT	UAUGUUGAGACCUGCGAGCTT
ITGB5-siRNA2	GCAAGUGCCAUGCAGGUUATT	UAACCUGCAUGGCACUUGCTT
ITGB5-siRNA3	GCUAUGAAAUGGCUUCAAATT	UUUGAAGCCAUUUCAUAGCTT
